# Supplementary material for: The Systems Biology Research Tool: evolvable open-source software
Source: BMC Syst Biol. 2008 Jun 29;2:55. doi: 10.1186/1752-0509-2-55 (PMC2446383; doi:10.1186/1752-0509-2-55)
Supplement: Additional file 1 — SBRT Archive. An archive of the current version of the Systems Biology Research Tool. [file 1752-0509-2-55-S1.zip › sbrt-1.4.0/doc/developers_guide/api/sbrt/shell/text/math/class-use/MathExprFormat.html]

Uses of Interface sbrt.shell.text.math.MathExprFormat


|  |  |  |
| --- | --- | --- |
| **Overview** \| **Package** \| **Class** \| **Use** \| **Tree** \| **Deprecated** \| **Index** \| **Help** | |  |
| PREV   NEXT | **FRAMES**    **NO FRAMES**     **All Classes** |  |


---


## **Uses of Interface sbrt.shell.text.math.MathExprFormat**

| Packages that use MathExprFormat | |
| --- | --- |
| **sbrt.shell.text.math** | Provides classes and interfaces for defining formats for mathematical objects. |

Uses of MathExprFormat in sbrt.shell.text.math

| Subinterfaces of MathExprFormat in sbrt.shell.text.math | |
| --- | --- |
| `interface` | `LinearCombFormat<V>`             This interface is used to represent formats for linear combinations. |

| Classes in sbrt.shell.text.math that implement MathExprFormat | |
| --- | --- |
| `class` | `LinearCombFormatV1<V>`             This class is a concrete implementation of `LinearCombFormat`. |
| `class` | `LinearCombFormatV2<V>`             This class is a concrete implementation of `LinearCombFormat`. |

---


|  |  |  |
| --- | --- | --- |
| **Overview** \| **Package** \| **Class** \| **Use** \| **Tree** \| **Deprecated** \| **Index** \| **Help** | |  |
| PREV   NEXT | **FRAMES**    **NO FRAMES**     **All Classes** |  |


---
